# Tissue Rigidity Increased during Carcinogenesis of NTCU-Induced Lung Squamous Cell Carcinoma In Vivo

**DOI:** 10.3390/biomedicines10102382

**Published:** 2022-09-23

**Authors:** Muhammad Asyaari Zakaria, Jazli Aziz, Nor Fadilah Rajab, Eng Wee Chua, Siti Fathiah Masre

**Affiliations:** 1 Centre for Toxicology and Health Risk Studies, Faculty of Health Sciences, Universiti Kebangsaan Malaysia, Kuala Lumpur 50300, Malaysia; 2 Department of Oral and Craniofacial Sciences, Faculty of Dentistry, University of Malaya, Kuala Lumpur 50603, Malaysia; 3 Centre for Healthy Ageing and Wellness, Faculty of Health Sciences, Universiti Kebangsaan Malaysia, Kuala Lumpur 50300, Malaysia; 4 Faculty of Pharmacy, Universiti Kebangsaan Malaysia, Kuala Lumpur 50300, Malaysia

**Keywords:** lung squamous cell carcinoma (SCC), carcinogenesis, pre-malignant, malignant, tissue rigidity, collagen, tenascin C (TNC), extracellular matrix (ECM)

## Abstract

Increased tissue rigidity is an emerging hallmark of cancer as it plays a critical role in promoting cancer growth. However, the field lacks a defined characterization of tissue rigidity in dual-stage carcinogenesis of lung squamous cell carcinoma (SCC) in vivo. Pre-malignant and malignant lung SCC was developed in BALB/c mice using *N-nitroso-tris-chloroethylurea* (NTCU). Picro sirius red staining and atomic force microscopy were performed to measure collagen content and collagen (diameter and rigidity), respectively. Then, the expression of tenascin C (TNC) protein was determined using immunohistochemistry staining. Briefly, all tissue rigidity parameters were found to be increased in the Cancer group as compared with the Vehicle group. Importantly, collagen content (33.63 ± 2.39%) and TNC expression (7.97 ± 2.04%) were found to be significantly higher (*p* < 0.05) in the Malignant Cancer group, as compared with the collagen content (18.08 ± 1.75%) and TNC expression (0.45 ± 0.53%) in the Pre-malignant Cancer group, indicating increased tissue rigidity during carcinogenesis of lung SCC. Overall, tissue rigidity of lung SCC was suggested to be increased during carcinogenesis as indicated by the overexpression of collagen and TNC protein, which may warrant further research as novel therapeutic targets to treat lung SCC effectively.

## 1. Introduction

Lung cancer is the most frequent type of cancer diagnosed worldwide. Lung cancer cases have affected 2.21 million individuals and caused 1.79 million deaths in 2020 [1]. Lung cancer was also the highest contributor to cancer-related deaths in 2017. The average five-year life expectancy of lung cancer patients in the year 2009 to 2015 was only 19%, which is the lowest compared with other types of cancer [2]. Lung cancer is a heterogeneous disease and can be categorized into small cell lung cancer (SCLC) and non-small cell lung cancer (NSCLC). The most commonly diagnosed lung cancer case is NSCLC which can be histologically divided into several subtypes such as adenocarcinoma, squamous cell carcinoma (SCC), large cell, and small cell [3]. Among these subtypes, the SCC subtype of lung cancer or lung SCC is gaining attention as it does not respond satisfactorily to the new treatments [4,5,6] and tends to develop resistance after treatment [7,8]. Therefore, a thorough understanding of lung SCC etiology and carcinogenesis is crucial to help cure the disease.

Cancer progression is commonly known to be accompanied by a dramatic increase in tissue rigidity. This condition was reported in various studies, which found an increased tissue rigidity in the breast [9], liver [10], brain [11], pancreas [12], and ovary [13] of cancer patients. For decades, this physical characteristic of cancer has been suggested as an emerging hallmark of cancer. Accumulating evidence has reported that the increment of tissue rigidity can trigger various biochemical signal activation through the mechanotransduction process at the molecular level, thus influencing the biological functions of cancer cells that promote cancer growth [14,15,16,17,18,19]. This remarkable feature of solid cancer is responsible for promoting cancer cell survival, proliferation, migration, invasion, and metastasis of cancer cells [9,16,20]. Clinical observation found that increased tissue rigidity is also associated with resistance to treatment, poor prognosis, and poor survival rate among cancer patients [17,21,22,23,24]. These findings proved the importance of tissue rigidity in promoting cancer growth. Therefore, reverting the increased tissue rigidity by targeting the factors contributing to increased tissue rigidity was suggested as a promising strategy for treating cancer.

Increased tissue rigidity is mainly contributed to by an increased overexpression, cross-linking, and deposition of extracellular matrix (ECM) proteins in the tumor microenvironment [25]. According to Burgstaller et al., lung cancer tissues present with an increased expression of various ECM proteins such as collagen, tenascin C, fibronectin, and laminin [26], which may contribute to increased tissue rigidity. Nevertheless, the characterization of tissue rigidity in lung cancer especially for the lung SCC subtype remains elusive as the previous study only performed tissue rigidity assessment in fibrotic lung tissues [27]. Moreover, the quantitative analysis of lung SCC tissue rigidity has not been comprehensively studied in vivo. Therefore, we aim to evaluate the tissue rigidity by analyzing the collagen (content, diameter, and rigidity) and tenascin C expression in NTCU-induced lung SCC tissues in mice models. This model was suggested as one of the best chemically-induced models that can closely mimic human lung SCC [28,29,30]. Collagen was chosen as it is the main component of ECM in cancer tissues. It plays a crucial role in sustaining the interstitial structure that affects physical tissue characteristics, including tissue rigidity [31,32]. Moreover, collagen can influence cancer cell behavior by interacting with other ECM molecules (i.e., laminin, fibronectin, and hyaluronic acid) and activating integrins, tyrosine kinase receptors, and some signaling pathways [31,33]. On the other hand, TNC is a hexameric glycoprotein that has been recently attributed to cancer growth. TNC can promote the transformation of fibroblasts to cancer-associated fibroblasts (CAFs), which are one of the primary sources of collagen [34,35,36]. Thus, TNC was also believed to stiffen the cancer tissues indirectly.

From this compelling evidence, we hypothesized that tissue rigidity might also be contributed to by increased deposition of collagen and TNC protein during lung SCC carcinogenesis. We believe that an increased understanding of the tissue rigidity by evaluating underlying factors that contribute to increased tissue rigidity may help researchers to understand the pathology of lung SCC better, thus providing essential knowledge for the development of effective therapy diagnosis, prognosis, and potential treatment for lung SCC in the future

## 2. Materials and Methods

### 2.1. Animal

Animal experiments were reviewed and approved by Universiti Kebangsaan Malaysia Animal Ethics Committee (FSK/2017/FATHIAH/24-MAY/846-MAY-2017-MAY-2020) and conducted in compliance with the ARRIVE guidelines. All procedures involving lung SCC animal model development were performed in our previous published work [28]. Briefly, lung SCC was established in female BALB/c mice using *N-nitroso-tris-chloroethylurea* (NTCU). The mice were assigned to Pre-malignant and Malignant groups. Then, the mice in each group were further divided into three groups (*n* = 6), which were Control (treated with 0.9% normal saline), Vehicle (treated with 70% acetone), and Cancer group (treated with 0.04 NTCU). As much as 25 µL of treatment was given to the dorsal area by skin painting, twice per week with a 3.5-day interval for 15 weeks (for Pre-malignant groups) and 30 weeks (for Malignant groups). After that, the mice were euthanized and the lungs were immediately harvested for the following procedures; picro sirius red (PSR) staining, atomic force microscopy, and immunohistochemistry (IHC) staining. Since we found no significant difference between the Control and Vehicle groups in our previous published work, we suggested that the Vehicle group alone is adequate as a valid control group for the respective assays. The proof of no significant difference between the Control and Vehicle groups was attached in the Supplementary File.

### 2.2. Picro Sirius Red (PSR) Staining

The paraffined embedded lung tissues were sectioned to 4 µm thickness on a glass slide. Then, the tissues were deparaffinized and rehydrated in a series of decreasing alcohol concentrations and tap water. After that, the tissues were fixed in Bouin’s solution for one hour at 60 °C, followed by staining in PSR (Sigma-Aldrich, Darmstadt, Germany) for one hour. Then, tissues were washed with 1% acetic acid (Chemiz, Shah Alam, Malaysia), followed by dehydration with alcohol and xylene before being mounted with Dibutylphthalate Polystyrene Xylene (DPX) (Sigma-Aldrich, Darmstadt, Germany) [37]. The average red staining percentage was measured using ImageJ 1.46r software equipped with Sirius Red Macro Plug-in (NIH, Bethesda, MD, USA) [38]. The measurement was performed on three different airways in each section and repeated for the other two sections to get a mean of the percentage of collagen red stain, representing collagen content from a minimum of three sections per mouse.

### 2.3. Atomic Force Microscopy

Freshly extracted lung tissues were rinsed with phosphate buffer saline and immediately embedded in FSC 22 (Leica Biosystems, Wetzlar, Germany) in preparation for cryosectioning. The cryofrozen tissues were sectioned to 10 µm thickness on glass slides using a cryostat (Leica CM1850 UV Leica Biosystems, Wetzlar, Germany). Then, the tissues were stored at 4 °C until further use for AFM. In this study, the AFM instrument used is JPK NanoWizard 3 AFM (JPK Instruments, Berlin, Germany). The cantilever used for imaging and force spectroscopy is a type of 0.01 nm paraboloid tip (Budget Sensors, Sofia, Bulgaria). The spring constant of the cantilever is 0.2 N/m, with a resonant frequency of 13 kHz. Cantilever calibration was performed by applying force spectroscopy using calibration manager mode on a clean glass slide. After that, the sensitivity was determined by fitting the linear slope of the contact region from the force curve. The thermal noise method was used to determine the cantilever’s spring constant. For imaging, the procedure was performed via contact mode and observed using two types of channels: error and height signal [39]. Firstly, an area of 100 µm^2^ was imaged to screen for collagen around airways. After an area of interest was found, smaller areas of collagen area were imaged at 20 µm^2^, 5 µm^2^, and 1 µm^2^. The 1 µm^2^ images were used to analyze the physical properties of collagen fibrils, including the diameter of fibrils and Young’s modulus. Fibril’s diameter was measured using JPK Data Processing software. Analysis was performed on three random collagen fibrils in each area of 1 µm^2^ to obtain a mean of the particular area. The measurement was repeated for the other 14 areas of 1 µm^2^ to get a final collagen diameter mean from 15 areas per mouse. Young’s modulus was calculated by fitting the contact region of the force curve using the Hertzian model. Analysis of Young’s modulus was performed on ten data points for each area of 1 µm^2^ to obtain a mean of collagen rigidity for the particular area. Similar to collagen diameter analysis, the measurement for Young’s modulus analysis was also performed at 15 areas of 1 µm^2^ to get the final mean which represents collagen rigidity for each mouse. Briefly, the sample (tissue) analyzed was suggested to possess an anisotropic and linearly elastic solid characteristic. Thus, the indenter was not deformable since no tip–sample interaction had occurred. Overall, the cantilever complies with Hooke’s Law, F = −kx, where F is the force applied by the spring, k is the spring constant of the cantilever, and x is the extension of the spring or deflection. Since a 0.01 nm paraboloid tip was used in this study, the following equation was utilized to calculate Young’s modulus:

(1) z=|(3kd−d0×1−v^2/1E √R|2/3+d−d0+Z0

where E is Young’s modulus, R is the tip radius, v is the Poisson ratio, d_0_ and Z_0_ are the corresponding values of cantilever deflection and the z-piezo extension at the contact point, respectively. Young’s modulus calculations were performed automatically by the JPK Data Processing software.

### 2.4. Immunohistochemistry (IHC) Staining

The paraffin-embedded lung tissues were cut into slices of 4 µm and placed on the charged slide. Initially, the tissues were deparaffinized at 60 °C in an oven for 30 min, followed by immediate transfer in xylene to remove the paraffin completely. Then, the tissues were rehydrated in absolute alcohol and phosphate buffer saline before incubating with antigen retrieval solution; 1 × 10 mM sodium citrate buffer (Merck, Darmstadt, Germany). After that, the tissues were incubated in 3% H_2_O_2_ (Merck, Germany) for 10 min to inhibit endogenous peroxidase activity, followed by incubation in 10% normal goat serum (NGS) (Dako, Glostrup, Denmark) for 10 min to block non-specific antibody binding sites. The tissues were then incubated with an anti-TNC antibody (Diluted to 1:50; Catalog No. T2551; Sigma-Aldrich, Darmstadt, Germany) overnight at 4 °C and then incubated with goat anti-rabbit horseradish peroxidase (Diluted to 1:100; Catalog No. PI-1000; Vector Laboratories, Newark, NJ, USA). In the negative control group, the tissues were incubated with 10% NGS only, without an anti-TNC primary antibody. After that, the IHC reactions on tissues were developed using the 3,3′-Diaminobenzidine (DAB) (Dako, Glostrup, Denmark) and counterstained with hematoxylin [28,40]. Finally, the tissues were dehydrated with alcohol and xylene before being mounted with DPX. The positive brown staining pixel intensity percentage was analyzed semi-quantitatively using FIJI-ImageJ 1.52p software (Java 8 version 64-bit) (NIH, Bethesda, MD, USA) [41].

### 2.5. Statistical Analysis

All data are presented as mean ± SEM and analyzed using Graphpad Prism version 8.3.0. The differences between groups were statistically analyzed using Student’s *t*-test for PSR staining, collagen content, collagen diameter, collagen rigidity, and IHC staining. A significant difference was assigned when the *p*-value was *p* ≤ 0.05.

## 3. Results

### 3.1. Collagen Content Increased in Lung SCC Tissues

**Figure 1:**
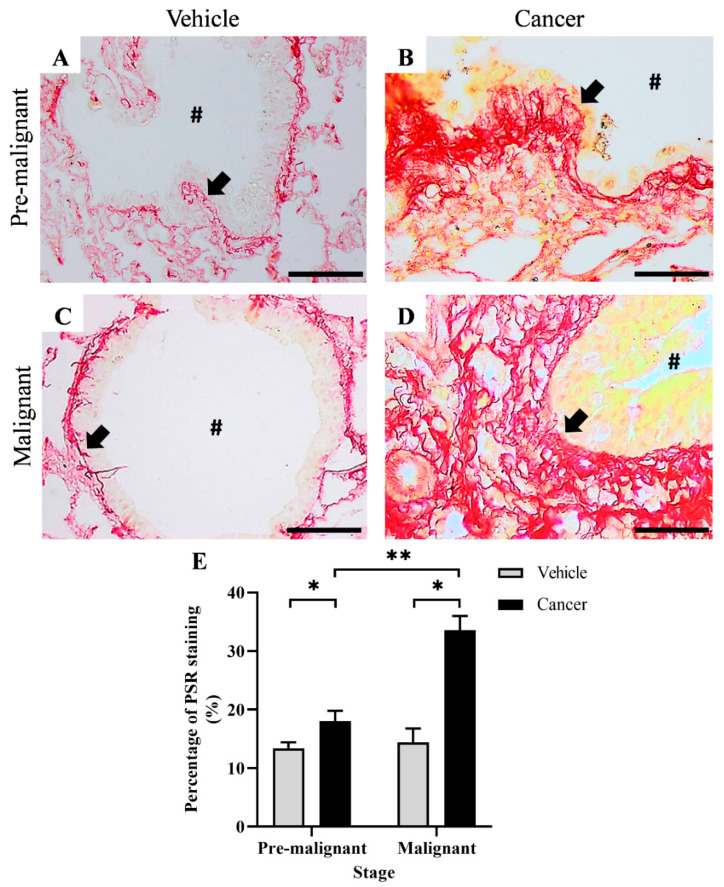
PSR staining at the bronchial area of the Vehicle and Cancer groups from both stages of carcinogenesis, at 40× magnification. (**A**,**C**) showed PSR staining of normal bronchial with thin collagen fibrils, (**B**) showed PSR staining of pre-malignant lung SCC tissues with increased collagen fibrils. (**D**) showed PSR staining of malignant lung SCC tissues with an increased and thick layer of collagen fibrils. (**E**) showed the percentage of the PSR staining in the Vehicle and Cancer groups from both stages of carcinogenesis. Data are presented as mean ± SEM. Arrows pointed to the collagen. **#** is the airway lumen. Scale bars = 50 µm. * is a significant difference (*p* < 0.05) between groups in the same stage. ** is a significant difference (*p* < 0.05) between Cancer groups from different stages of carcinogenesis. *p* < 0.05 using Student’s *t*-test.

Figure 1A–D shows the PSR staining, which stains collagen as red around the bronchi of lung tissues obtained from the Pre-malignant and Malignant stage at 40× magnification. Figure 1A,B show the PSR staining from the Vehicle and Cancer groups of the Pre-malignant stage, respectively. Figure 1C,D show the PSR staining from the Vehicle and Cancer groups of the Malignant stage, respectively. Generally, the red staining was observed to be higher and more abundant in the Cancer groups (Figure 1B,D) as compared with the Vehicle groups (Figure 1B,D) from both stages of carcinogenesis

Figure 1E shows the bar chart for the percentage of PSR staining (%), which indicates collagen content. Briefly, the Pre-malignant Cancer group was found to have a significantly higher (*p* < 0.05) collagen content of 18.08 ± 1.75% as compared with the Pre-malignant Vehicle group; 13.41 ± 1.01%. Likewise, the Malignant Cancer group was found to have a significantly higher (*p* < 0.05) collagen content, which is 33.63 ± 2.39% as compared with the Malignant Vehicle group; 14.39 ± 2.38%. A significant difference in collagen content between Cancer groups from different stages of carcinogenesis was also observed in this study, where the Malignant Cancer group was found to have a significantly higher (*p* < 0.05) collagen content as compared with the Pre-malignant Cancer group.

### 3.2. Collagen Structure Disrupted in Lung SCC Tissues

**Figure 2:**
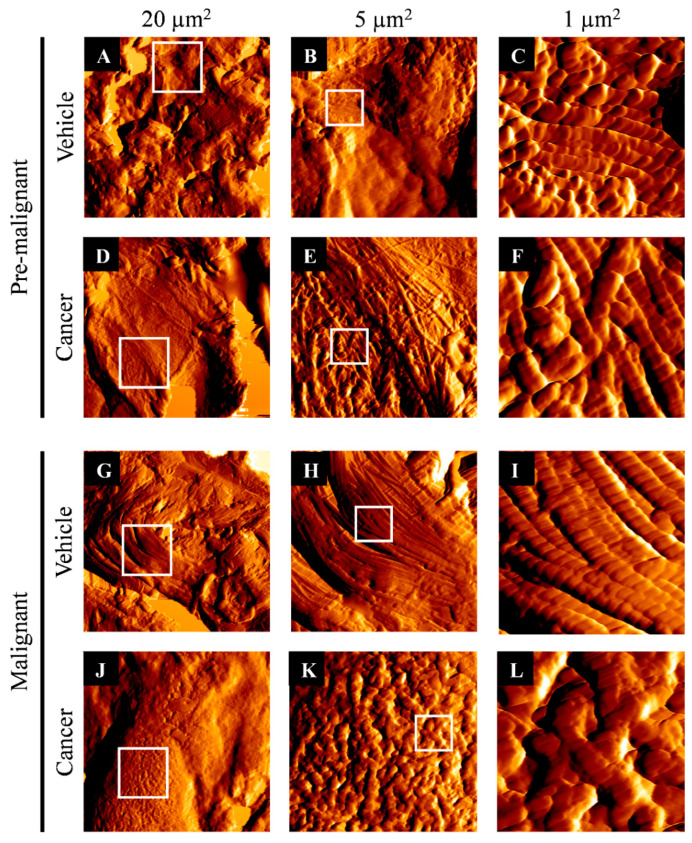
Collagen fibrils at the bronchial area of the Vehicle and Cancer groups from both stages of carcinogenesis, in an area of 20 µm^2^, 5 µm^2^, and 1 µm^2^. (**A**–**C**) show the images of collagen fibrils from the Pre-malignant Vehicle group. (**D**–**F**) show the images of collagen fibrils from the Pre-malignant Cancer group. (**G**–**I**) show the images of the Malignant Vehicle group. (**J**–**L**) show the images of collagen fibrils from the Malignant Cancer group. The white border-box is the area selected for the subsequent magnified image scans using atomic force microscopy.

Figure 2A–L shows the collagen fibrils that were imaged around the bronchi of lung tissues, obtained from the Pre-malignant and Malignant stages at an area of 20 µm^2^, 5 µm^2^, and 1 µm^2^. Figure 2A–C,G–I show normal collagen fibrils that were imaged from the Vehicle groups of the Pre-malignant and Malignant stages, respectively. The normal collagen fibrils in the area of 1 µm^2^ from the Vehicle groups can be observed as an elongated string of a consistently sized ‘pearl-necklace’, which was orderly arranged without cross-linking with each other. Meanwhile, Figure 2D–F,J–L show collagen fibrils that were imaged from the Cancer groups of the Pre-malignant and Malignant stages, respectively. The collagen fibrils in the Pre-malignant Cancer group were found to have some cross-linking with the inconsistency of collagen fibril size and arrangement. Likewise, the structure of collagen fibrils in the Malignant Cancer group was found to be highly cross-linked without an orderly arrangement of collagen. Briefly, the Malignant Cancer group (Figure 2L) shows a more prominent disruption of collagen structure than the Pre-malignant Cancer group (Figure 2F) and the Vehicle groups from both stages of carcinogenesis (Figure 2C,I).

### 3.3. Collagen Diameter Remains Same in The Normal and Lung SCC Tissues

**Figure 3:**
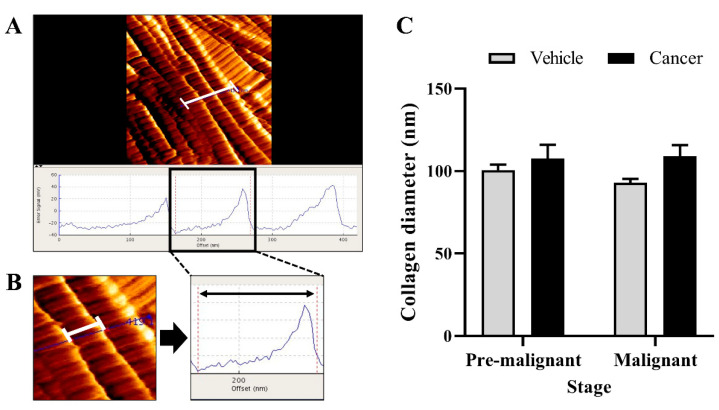
(**A**) The collagen diameter measurement for three collagen fibrils with their respective micrograph scale bar. (**B**) The magnified collagen diameter measurement for one collagen fibril with their respective micrograph scale bar. The double-sided arrow shows the length of one collagen diameter measured. (**C**) The bar chart of collagen diameter (nm) at the bronchial area of the Vehicle and Cancer groups from both stages of carcinogenesis. Data are presented as mean ± SEM.

Figure 3 shows the result for collagen diameter measured at the bronchial area of the Vehicle and Cancer groups, obtained from the Pre-malignant and Malignant stages. Based on the bar chart in Figure 3C, no significant difference in the collagen diameter was observed between the Vehicle and Cancer groups from both stages of carcinogenesis. However, the collagen diameter in the Cancer groups from the Pre-malignant and Malignant stages was found to be wider, which are 107.7 ± 8.2 nm and 109 ± 6.82 nm, respectively, as compared with the Vehicle groups from the Pre-malignant (100.5 ± 3.45 nm) and Malignant stage (92.91 ± 2.39 nm).

### 3.4. Collagen Rigidity Increased in the Lung SCC Tissues

**Figure 4:**
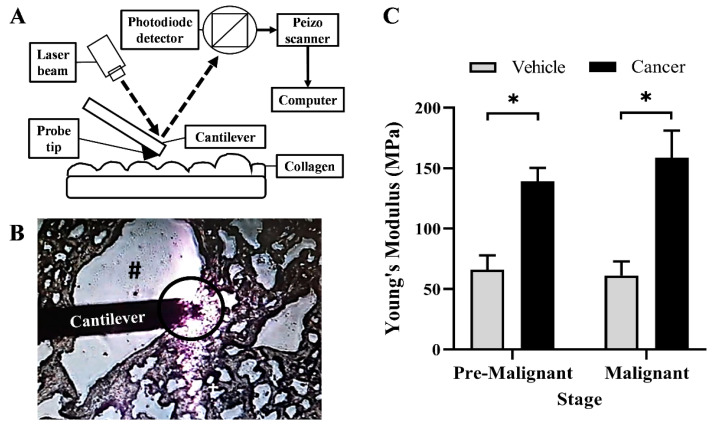
(**A**) The components of the standard atomic force microscopy that works by reflecting the laser focused on the cantilever, which is then captured by the scanner and measured as Young’s modulus by the computer software. (**B**) The aerial view of the working cantilever at a bronchial area where the measurement of collagen rigidity was taken. # shows the bronchial airway. The circle shows the area of the probe tip, which measured the collagen rigidity. (**C**) The bar chart of Young’s modulus (MPa) at the bronchial area of the Vehicle and Cancer group from both stages of carcinogenesis. Data are presented as mean ± SEM. * is a significant difference (*p* < 0.05) between the Vehicle and Cancer groups in the same stage of carcinogenesis. *p* < 0.05 using Student’s *t*-test.

Figure 4 shows the result for collagen rigidity at the bronchial area of the Vehicle and Cancer groups, obtained from the Pre-Malignant and Malignant stages. Based on the bar chart in Figure 4C, for the Pre-malignant stage, the Cancer group was found to have a significantly higher (*p* < 0.05) Young’s modulus of 139 ± 11.3 MPa as compared with the Vehicle group; 66.01 ± 11.9 MPa. The same difference was also observed for the Malignant stage, in which the Cancer group shows a significantly higher (*p* < 0.05) Young’s modulus of 158.8 ± 22.2 as compared with the Vehicle group; 61.23 ± 11.76 MPa. Notably, this study found no significant difference (*p* < 0.05) in Young’s modulus between the Cancer groups from two different stages of carcinogenesis.

### 3.5. Tenascin-C Expression Increased in Lung SCC Tissues

**Figure 5:**
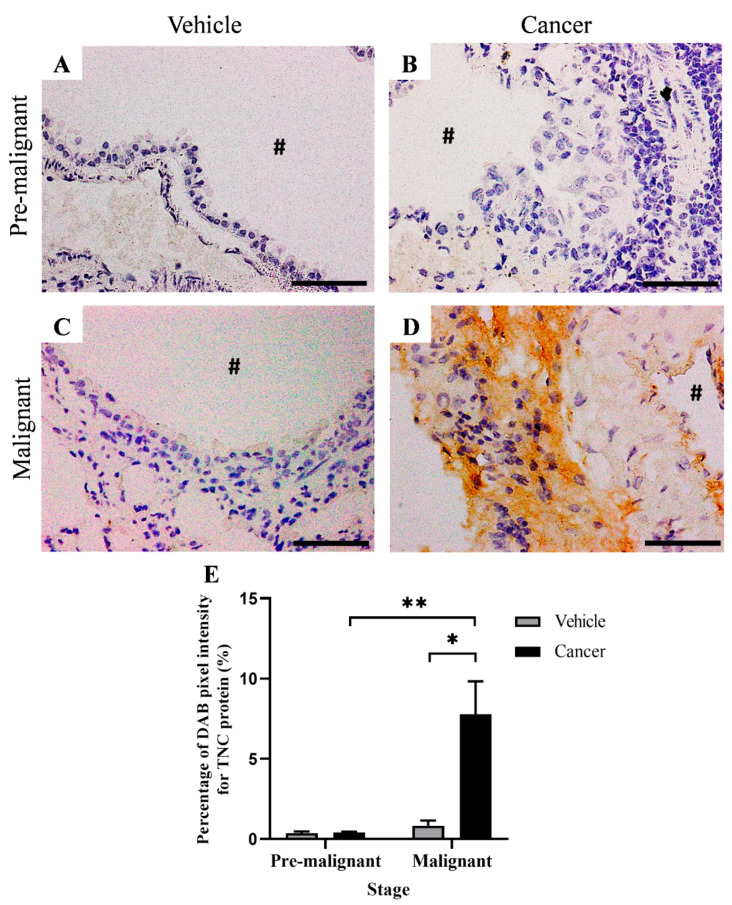
Immunohistochemistry (IHC) staining for TNC protein at the bronchial area of the Vehicle and Cancer groups from both stages of carcinogenesis, at 40× magnification. (**A**,**C**) showed IHC staining of normal bronchial (**B**) showed IHC staining of Pre-malignant lung SCC tissue with no observable DAB brown staining. (**D**) showed IHC staining of Malignant lung SCC tissue with an observable DAB brown staining at the extracellular matrix around the bronchial. (**E**) showed the percentage of the IHC staining in the Vehicle and Cancer groups from both stages of carcinogenesis. Data are presented as mean ± SEM. # is the airway lumen. Scale bars = 50 µm. * is a significant difference (*p* < 0.05) between groups in the same stage. ** is a significant difference (*p* < 0.05) between Cancer groups from different stages of carcinogenesis. *p* < 0.05 using Student’s *t*-test.

Figure 5 shows the IHC staining for TNC protein at the bronchial area of the Vehicle and Cancer groups, obtained from the Pre-malignant and Malignant stages. Based on the histology observation, positive IHC staining (DAB brown staining) for TNC protein was not observed in the Pre-malignant Vehicle (Figure 5A) and Malignant Vehicle (Figure 5C) groups. Likewise, TNC expression was also not observed in the Pre-malignant Cancer group (Figure 5B). Notably, the TNC protein expression was only observed in the Malignant Cancer group in the extracellular matrix (Figure 5D).

Figure 5E shows the bar chart of the percentage of DAB pixel intensity for TNC protein. The Malignant Cancer group was found to have a significantly higher (*p* < 0.05) percentage of DAB pixel intensity for TNC protein, which is 7.97 ± 2.04% as compared with the Malignant Vehicle group; 0.84 ± 0.31%. Additionally, the percentage of DAB pixel intensity for the TNC protein in the Malignant Cancer group was also found to be significantly higher (*p* < 0.05) as compared with the Pre-malignant Cancer group; 0.45 ± 0.53%.

## 4. Discussion

For decades, tissue rigidity has been clinically associated with various pathological conditions including cancer [42,43,44]. Compelling evidence shows that this physical characteristic of cancer increases during carcinogenesis and is responsible for promoting multiple hallmarks of cancer, which stand this physical characteristic as important as genetic/epigenetic alterations in cancer [20,45,46,47,48,49]. Considering their function, the characterization of tissue rigidity in lung SCC, focusing on its underlying contributor is crucial to understand the disease better.

Increased tissue rigidity was contributed to by multiple components, mainly by over-synthesis and deposition of ECM proteins [16]. Among the ECM proteins, collagen is the most well-known protein to be strongly associated with tissue rigidity as it plays a principal role in maintaining the integrity and architecture of the tissue [31]. Therefore, the dysregulation in collagen homeostasis during cancer growth such as collagen over synthesis, as observed in breast [50], pancreas [51], and lung cancer [26], can significantly increase the rigidity of tissue. Based on the PSR staining, the collagen content was found to be more abundant and increased as cancer progressed from the Pre-malignant stage to the Malignant stage of lung SCC. This finding agrees with previous studies that reported increased collagen content in the 3D culture containing NSCLC cell line [52] and in various stages of lung SCC tissues obtained from the clinical setting [53]. Collagen is one of the important ECM proteins that make up the basement membrane, which underlies the bronchial epithelial cells [26,54]. According to Yurchenco [54] and Gatseva et al. [55], the basement membrane comprises multiple types of collagen such as collagen types 4, 6, 7, 15, 17, and 18. Thus, it is not surprising to observe a variably thickened basement membrane in the Pre-malignant and Malignant Cancer groups due to the presentation of more abundant collagen fibrils, which was further confirmed by the quantitative analysis of collagen content.

Analysis of collagen structure and diameter is a sub-analysis that can be analyzed using atomic force microscopy. Briefly, the collagen structure in the Cancer group was found to intersect with each other and was observed to be more prominent in the Malignant stage than in the Pre-malignant stage. According to Xiao and Ge [56], the cross-linking between collagen was induced by the lysyl oxidase enzyme, which significantly increased tissue rigidity in cancer. Parallel to the observations of collagen structure from this study, increased cross-linking between collagen has also been reported by Navab et al. [57] and Cox et al. [58] in the NSCLC xenophobic tissues and lung cancer of mice model, respectively. They also concluded that the increase in cross-linking between collagen is strongly associated with the rigidity of lung cancer tissues. According to Liu et al., collagen cross-linking present at the metastatic site can increase therapeutic resistance as tissue rigidity increases [59]. Collagen cross-linking is also primarily associated with angiogenesis, suggesting the critical role of collagen cross-linking in favoring cancer growth [32]. For collagen diameter analysis, our study found no significant difference between cancerous and normal tissues. Our finding agrees with a prior study by Achilli et al. [60] that reported that collagen diameter was not affected by increased collagen concentration and density. Although insignificant, cancer tissue was found to have a slightly higher collagen diameter than normal tissues for both stages of carcinogenesis. As the collagen was highly dysregulated and over-synthesized in the cancer microenvironment, the density of collagen fibrils was also increased [61], which may be the reason for a slight increase in the diameter of collagen fibrils in the Cancer groups from both stages of carcinogenesis. This finding contrasts with recent studies that reported a reduction in collagen diameter in idiopathic tissues pulmonary fibrosis, a pathological condition that can increase the risk of developing lung cancer [62,63]. Despite the contrary findings, the analysis of collagen structure and diameter was only rated as a supporting determinant for tissue rigidity in our study because it was unable to reflect the actual tissue rigidity as evaluated through collagen fibril bending stiffness or collagen rigidity [64].

Collagen rigidity is one of the main analyses that can be assessed by using an atomic power microscope. The rigidity evaluated in the study did not measure the overall rigidity of tissues that include cells, connective tissues, vascular walls, and other components because we only consider the value of the rigidity of collagen fibrils. Therefore, the value measured is higher so that it reaches the value of MPa units as compared with the analysis of whole lung tissues, which is in the range of kPa units. The value of collagen rigidity that hits MPa units is normal considering that previous studies have also reported that Young’s modulus values of cultured fibrils collagen type 1 and normal mice tail collagen fibrils are about 70 MPa [65] and 11–95 MPa [66], respectively. Generally, this study found that the cancerous tissues had a higher collagen rigidity of about 139 MPa for the Pre-malignant stage and 159 MPa for the Malignant stage, as compared with normal tissues with a Young’s modulus value of about 61–66 MPa. The increase in collagen rigidity in lung SCC matched the pattern of discoveries made by F. Liu et al. [67] and Booth et al. [27], who reported an increase in the rigidity of fibrosis lung tissues; a pathological condition that resembles lung cancer tissues in terms of collagen deposition. They found that the fibrotic lung has a Young’s modulus value in the range of 2–35 kPa, which is higher than normal lungs that have a reading value of Young’s modulus of about 0.2–2 kPa. From this study, we can observe the insignificant increment of collagen rigidity from the Pre-malignant to the Malignant stage, which may result from the plateau of collagen rigidity as the cancer tissue progress to the malignant stage. However, we still can appreciate an increased trend of collagen rigidity as cancer progress, though no significant difference was identified. The increment trend agrees with previous studies that reported a significant increase in tissue rigidity during breast cancer carcinogenesis [9,68] and hepatocellular carcinoma [69], which measure the rigidity of the whole tissues. To the best of our knowledge, this study is the first to determine the rigidity of lung SCC tissues by quantitatively measuring collagen rigidity at the pre-malignant and malignant stages.

Apart from collagen, another parameter assessed to determine tissue rigidity is the expression of TNC. TNC is an essential multimodular glycoprotein of ECM, which can bind to soluble factors and matrix components [70]. It is among the most upregulated ECM protein found in the ECM of primary lung cancer as compared with the normal tissue [26,71]. TNC was produced by both the stromal and cancer cells, which will eventually promote cancer cell survival and growth by remodeling the ECM [72]. As a result, the ECM-nourished cancer cells can increase the secretion of more TNC, thereby upregulating the TNC expression in a positive feedback manner during carcinogenesis [34]. Notably, the crosstalk between TNC and ECM in lung SCC is still in its infancy. However, the studies on other types of cancer such as breast, brain, and colorectal cancer, did provide remarkable insight into the function of TNC on cancer growth [34,73,74,75]. Mechanistically, TNC can bind with the integrin and activate several signaling pathways to facilitate the oncogenic events. For instance, TNC can attach to the αvβ1 and αvβ6 integrins, thereby facilitating the epithelial-mesenchymal-transition that promotes migration in breast cancer cells [76]. Additionally, TNC can activate mTOR and NOTCH signaling pathways to promote chemotherapy resistance in breast cancer and sphere formation in brain cancer, respectively [75,77]. Overall, these findings indicate that TNC may play a crucial role in promoting cancer growth, suggesting it as an interesting target for future functional studies, especially in lung SCC. From this study, we only found an increased expression of TNC in the malignant-grade lung SCC cancer tissues. Our finding is in line with a previous study conducted by Rzechonek et al., who found increased TNC in the more advanced stage of both adenocarcinoma and SCC subtypes of lung cancer tissues [78]. Its expression in the final stages may be due to its prominent role associated with malignant phenotypes such as supporting cell migration, angiogenesis, and protecting cells from being killed by immune cells during metastasis [79,80,81]. Moreover, TNC has been shown to be significantly associated with larger tumor size and lung cancer metastasis involving lymph nodes [70,71,82]. Specifically, TNC promotes metastasis by reducing cellular adhesion and supporting the survival of migrating cancer cells to the secondary site [73,83], suggesting this ECM protein as a potential biomarker for lung cancer prognosis. TNC can also change the physical properties of cancer tissues, particularly by increasing their rigidity [34]. Based on the transcriptomic analysis conducted by Schiller et al. [84], an increased TNC protein in the ECM showed a high association with lung tissue rigidity. This relationship is supported by Katoh et al. [34], who report TNC involvement directly in carrying out the transformation of fibroblasts to CAFs, which is one of the primary sources of collagen. In summary, collagen content rated higher in the Malignant stage of lung SCC, as observed in this study, which was likely contributed to by the significantly higher and exclusive TNC protein expression. As a result, malignant-grade lung SCC tissues become more rigid due to increased collagen content.

For future studies, it may be interesting to determine the effect of collagen and TNC knock-down on tissue rigidity in NTCU-induced lung SCC in vivo, considering the essential role of both ECM proteins in sustaining tissue rigidity. Moreover, cancer progression is also worth evaluating following collagen and TNC knock-down.

## 5. Conclusions

Briefly, our results suggested that the lung SCC is characterized by increased tissue rigidity as indicated by increased collagen content, collagen rigidity, and TNC expression from the pre-malignant to the malignant stage. Thereby, the factors contributing to increased tissue rigidity as analyzed in this study were suggested as promising targets to treat lung SCC more effectively in the future.

## Data Availability

All data generated during this study are included in this published article and Supplementary File.

## Supplementary Materials

The following supporting information can be downloaded at: https://www.mdpi.com/article/10.3390/biomedicines10102382/s1. Figure S1: **H&E Staining**; Figure S2: **Epithelium thickness**.
