# Supplementary material for: Tissue Rigidity Increased during Carcinogenesis of NTCU-Induced Lung Squamous Cell Carcinoma In Vivo
Source: Biomedicines. 2022 Sep 23;10(10):2382. doi: 10.3390/biomedicines10102382 (PMC9598693; doi:10.3390/biomedicines10102382)
Supplement: Supplementary file 1 [file biomedicines-10-02382-s001.zip › biomedicines-1898756-supplementary.pdf]

Proof of no significant difference between the Control and Vehicle groups at both stages of carcinogenesis was observed from the Hematoxylin and Eosin (H&E) staining, histopathology scoring, and epithelium thickness measurement. These assays were perfomed in our previous published work [1].

1. **H&E Staining**
   From the H&E staining shown below, we can observe a single layer of epithelial cells (point by arrows) at the bronchial area in the Control and Vehicle groups at both stages of carcinogenesis **(Dashed box)**. The single-layer epithelium indicated normal histology of bronchial and lung tissues. * represent airway. Scale bar = 50 µm.

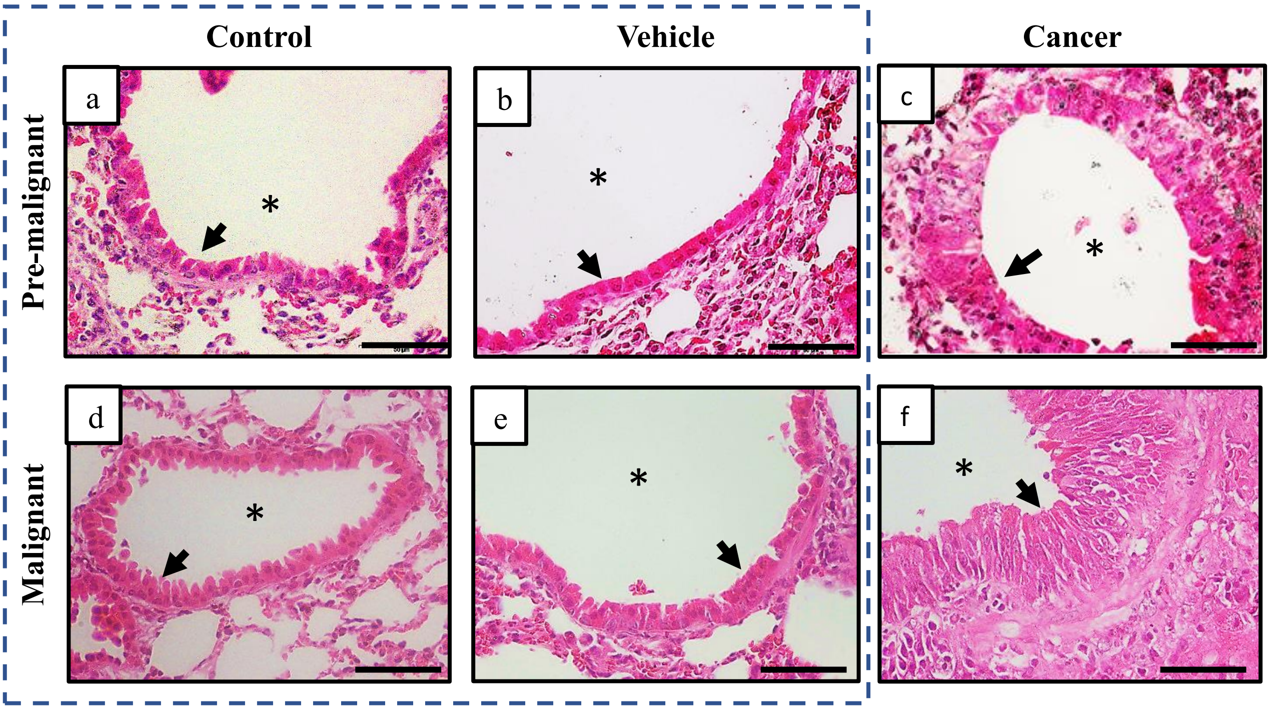

Figure S1. H&E Staining.

2. **Histopathology scoring**
   We did score the lung tissue according to the histology characteristics of squamous cell carcinoma as follows: Normal=0; Hyperplasia=1; Metaplasia=2; Dysplasia=3; Carcinoma in situ/invasive=4 [2,3]. From our observation, we found that both the Control and Vehicle groups at both stages of carcinogenesis obtained the same cumulative mean of 0, indicating normal histology of bronchial and lung tissues. Whereas, for the Cancer groups, the score is 2.00 ± 0.29 (Combination of Hyperplasia, Metaplasia, and Dysplasia) and 4.00 ± 0.00 (Carcinoma in situ/invasive) at the Pre-malignant and Malignant stages, respectively.

3. **Epithelium thickness**
   Based on the H&E staining, we measured the epithelium thickness as it is one of the grading criteria for the pulmonary lesion. Generally, we found no significant difference between the Control and Vehicle groups at both stages of carcinogenesis, as shown in the figure below **(Dashed box)**. At the Pre-malignant stage, the epithelium thickness for the Control and Vehicle groups are 11.05 ± 0.23 µm and 11.53 ± 0.35 µm,

respectively. At the Malignant stage, the epithelium thickness for the Control and Vehicle groups are 11.54 ± 0.33 µm and 10.86 ± 0.34, respectively. Whereas the epithelium thickness for cancer groups are 20.72 ± 1.27 µm and 39.71 ± 2.27 at the Pre-malignant and Malignant stages, respectively.* is the significant difference between Control and Vehicle groups with Cancer groups in the same stage of carcinogenesis. ** is the significant difference between Cancer groups at two different stages of carcinogenesis.

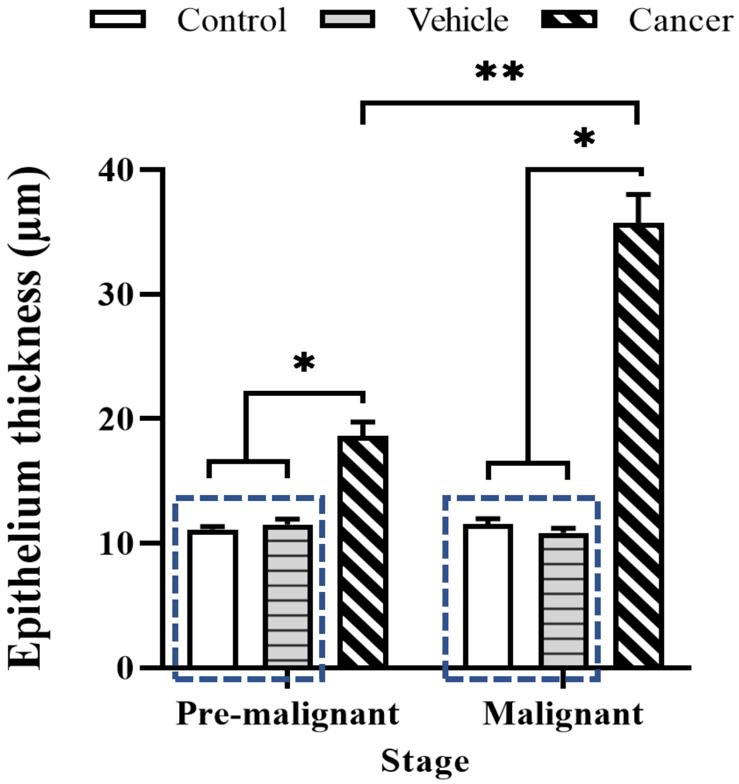

Figure S2. **Epithelium thickness**.

References

1. Zakaria, M.A.; Rajab, N.F.; Chua, E.W.; Selvarajah, G.T.; Masre, S.F. NTCU induced pre-malignant and malignant stages of lung squamous cell carcinoma in mice model. *Sci. Rep.* **2021**, *11*, 22500.

2. Surien, O.; Ghazali, A.R.; Fathiah Masre, S. Histopathological effect of pterostilbene as chemoprevention in N-NITROSO-TRI-CHLOROETHYLUREA (NTCU)-Induced lung squamous cell carcinoma (SCC) mouse model. *Histol. Histopathol.* **2020**, *35*, 1159-1170.

3. Wang, Y.; Zhang, Z.; Garbow, J.R.; Rowland, D.J.; Lubet, R.A.; Sit, D.; Law, F.; You, M. Chemoprevention of Lung Squamous Cell Carcinoma in Mice by a Mixture of Chinese Herbs. *Cancer Prev. Res.* **2009**, *2*, 634–641.
